# Immunological Aspects of X-Linked Chronic Granulomatous Disease Female Carriers

**DOI:** 10.3390/antiox10060891

**Published:** 2021-06-01

**Authors:** Maria Chiriaco, Irene Salfa, Giorgiana Madalina Ursu, Cristina Cifaldi, Silvia Di Cesare, Paolo Rossi, Gigliola Di Matteo, Andrea Finocchi

**Affiliations:** 1 Department of Systems Medicine, University of Rome Tor Vergata, via Montpellier 1, 00133 Rome, Italy; giorgiana.mada@gmail.com (G.M.U.); di.cesare@med.uniroma2.it (S.D.C.); rossipa@med.uniroma2.it (P.R.); di.matteo@med.uniroma2.it (G.D.M.); 2 Research Unit of Primary Immunodeficiency, Academic Department of Pediatrics, Immune and Infectious Diseases Division, Bambino Gesù Children Hospital, Piazza S. Onofrio 4, 00165 Rome, Italy; irene.salfa@gmail.com (I.S.); cristina.cifaldi@gmail.com (C.C.)

**Keywords:** chronic granulomatous disease (CGD), X-linked CGD carrier, reactive oxygen species (ROSs), nicotinamide dinucleotide phosphate oxidase (NADPH), dihydrorhodamine (DHR) assay, X-chromosome inactivation (XCI), immune dysregulation

## Abstract

X-linked Granulomatous Disease (XL-CGD) carriers were previously thought to be clinically healthy because random X-chromosome inactivation (XCI) allows approximately half of their phagocytes/monocytes to express functional gp91phox protein. This supports the NADPH oxidase activity necessary for the killing of engulfed pathogens. Some XL-CGD carriers suffer from inflammatory and autoimmune manifestations as well as infections, although the skewed-XCI of a mutated allele is reported to be exclusively determinant for infection susceptibility. Indeed, immune dysregulation could be determined by dysfunctional non-phagocytic leukocytes rather than the percentage of functioning neutrophils. Here we investigated in a cohort of 12 X-CGD female carriers at a particular time of their life the gp91phox protein expression/function and how this affects immune cell function. We showed that 50% of carriers have an age-independent skewed-XCI and 65% of them have a misrepresented expression of the wild-type gene. The majority of carriers manifested immune dysregulation and GI manifestations regardless of age and XCI. Immunological investigations revealed an increase in CD19+ B cells, CD56bright-NK cell percentage, a slightly altered CD107a upregulation on CD4+ T cells, and reduced INFγ-production by CD4+ and CD8+ cells. Notably, we demonstrated that the residual level of ROS robustly correlates with INFγ-expressing T cells, suggesting a role in promoting immune dysregulation in carriers.

## 1. Introduction

Chronic granulomatous disease (CGD) is the most common inherited disorder of phagocytes caused by genetic defects in genes encoding the nicotinamide dinucleotide phosphate (NADPH) oxidase subunits, essential for the clearance of phagocytized pathogens by *respiratory burst* mechanisms [1]. X-linked CGD (X-CGD) results from mutations in the *CYBB* gene encoding the gp91phox subunit (about 70% of patients), while autosomal recessive (AR) CGD is associated with mutations in one of the *CYBA*, *NCF1*, *NCF2,* and *NCF4* genes encoding for p22phox, p47phox, p67phox, and p40phox respectively. Recently, a new AR-CGD caused by mutations in the *CYBC1* gene, characterized by a complete absence of CYBC1 protein and severe reduction of gp91phox protein expression, was described [2]. The inability to produce reactive oxygen species (ROSs) leads CGD patients to be highly susceptible to life-threatening bacteria and fungal infections and granuloma formation in several organs, including gastrointestinal-, genitourinary- and skin-tract. Moreover, the unresolved chronic inflammation predisposes patients to significant immune dysregulation, often presenting with autoimmune phenomena such as Systemic- and Discoid- Lupus Erythematosus, thrombocytopenic purpura, arthritis, and inflammatory bowel disease (IBD) [3,4]. Patients with X-CGD usually show a more severe clinical phenotype with disease onset at an earlier age.

The X-linked CGD *carrier status* is easy to identify in females because they carry one copy of the mutated gene and show two populations of neutrophils, one positive and one negative for gp91phox expression and function [5,6]. It was traditionally thought that female carriers of X-linked recessive diseases are clinically healthy on account of a stochastic model of X-chromosome inactivation (XCI) that occurs randomly for one of the two X chromosomes in their cells during development. Indeed, female X-CGD carriers are usually asymptomatic because their neutrophils are able to produce a sufficient level of ROSs to support the killing of pathogens. Currently, several factors, including certain mutations and the woman’s age, seem to influence the choice of X chromosome for inactivation, allowing a non-random (skewed) XCI that causes clinical manifestations of the disease [7]. Indeed, X-CGD carriers may develop a variety of symptoms, comprising IBD and autoimmunity, depending on the outcome of XCI through which the non-functional gp91phox protein, encoded by the mutated allele, takes over [8,9]. In order to effectively assess the risk of infections in X-CGD carriers, it is important to evaluate the residual level of ROSs produced by the NADPH oxidase complex. In particular, it has been shown that X-CGD carriers presenting with functioning neutrophils ranging from 5% to 30% are prone to have infections. In contrast, the susceptibility to autoimmune/inflammatory manifestations in the *carrier’s status* is not clearly correlated to the amount of baseline oxidative burst function [10,11,12].

In view of these findings, we studied a cohort of 12 X-CGD female carriers at a particular time of their life through a retrospective chart review of their clinical manifestations, analyzing the gp91phox protein expression/function and how this affects the function of immune cells. Our results showed that: (a) the percentage of NADPH oxidase activity correlates with the expression of the wild-type (wt) allele; (b) the 50% of carriers have a skewed-XCI, regardless of carriers’ age; (c) the majority of skewed XCI carriers presents the mutated *CYBB* allele; (d) most carriers manifested immune dysregulation and GI manifestations; (e) carriers show an increased percentage in CD56bright NK cells, a slightly altered CD107a upregulation on T cell and a reduced INFγ-production by CD4+ and CD8+ cells and (f) finally we demonstrate that in X-CGD carriers the residual level of ROS produced by the NADPH oxidase complex is robustly correlated with INFγ cytokine production by T cells.

## 2. Material and Methods

### 2.1. Patients and Informed Consent

In this study, fifteen females related to X-CGD patients who are followed by our Center for the follow-up were enrolled. All procedures performed in the study were in accordance with the ethical standards of the institutional research committee and with the 1964 Helsinki declaration and its later amendments or comparable ethical standards. Informed consent, following standard ethical procedures with approval from the Children’s Hospital Bambino Gesù Ethical Committee (Prot. n. 80 CM/vp), was obtained from each subject. Both HDs (*n* = 25) and females enrolled (*n* = 15) were free of infection and were off therapeutic treatment. The three females found without mutations were considered healthy and used as normal control to have a total of 28 HDs with age ranging from 25 to 59 years old. Clinical information was retrieved from interviews, and molecular data of all investigated subjects are summarized in Table 1. X-CGD carriers (*n* = 12) were divided by age into (A) young adults (age 20–39 years, *n* = 4) and (B) middle-aged adults (age 40–59 years, n = 8) and further sub-groups ‘symptomatic and asymptomatic’, were used in consideration of symptoms.

### 2.2. Clinical Features

Female carriers were considered symptomatic when presenting with *infections* (urogenital, urinary, and gastrointestinal), *immune dysregulation* (aphthous stomatitis, cutaneous eczema, psoriasis, and discoid lupus), and *gastrointestinal (GI) manifestation* (colitis/ulcerative colitis, gastric disease, and chronic diarrhea). Female carriers without any abovementioned symptoms and/or with non-CGD-specific features (breast/reproductive tract diseases, dental/eye diseases, and allergy) were considered asymptomatic.

### 2.3. Molecular and Biochemical Investigations

Sanger sequencing analysis revealed a mutation in the *CYBB* gene using the protocol described in Di Matteo G et al. [13]. All mutations were confirmed by the gp91phox protein investigations (FACS) and NADPH-oxidase function (DHR-assay by FACS) as described in Chiriaco M et al. [14]. Neutrophils from female X-linked carriers of CGD showed two populations of cells expressing or not the gp91phox protein (gp91− or gp91+), allowing a functional or not NADPH oxidase complex (DHR− or DHR+). X-chromosome inactivation (XCI) was considered ‘random’ when the % of DHR+ cells ranged from 25 to 75 [15] and ‘non-random or skewed’ when it is outside of 25:75.

### 2.4. Flow-Cytometry Studies

*Immune-phenotype*: cells were stained with antibodies against anti-human gp91phox-FITC (anti-flavocytochrome B558, clone 7D5) (MBL, Medical and Biological Laboratories Co, Japan), CD3-PERCP (BD), CD4-APC (BD), CD8-PE (BD), CD19-APC (BD), CD56-PE (BD) and CD16-FITC (BD) for lymphocytes, CD16-PECY7 (BD) and CD14-APC (BD) for monocytes. Cells were incubated with the appropriate antibody cocktail for 30 min at 4 °C, then washed with PBS and analyzed by FACS. *DHR assay*: the capacity of neutrophils to produce ROS via the NADPH oxidase was investigated by the Phagoburst ^TM^ kit according to the manufacturer’s instructions. *CD107a degranulation Assay*: PBMCs were isolated by density-gradient centrifugation with Ficoll-Paque PLUS (GE Healthcare) according to the manufacturer’s protocol. To study the NK (CD56+) and NK-T like (CD3+CD56+) cells, PBMC from subjects and HDs were plated in complete medium (RPMI-1640, 10% FBS, glutamine, penicillin, and streptomycin) and stimulated overnight (o.n.) with IL2 plus PMA (6h) and then stained with CD107-APC (BD), CD3-PERCP (BD), and CD56-PE (BD) for 30 min at 4 °C, then washed with PBS and analyzed by FACS. To study CD4+ and CD8+ T cells, PBMC cultured in complete medium were stimulated with OKT3 (o.n) plus PMA (6h) and then stained with CD4-APC (BD), CD8-PE (BD) for 30 min at 4 °C and then washed in PBS. For the study of INFγ, Brefeldin A (500X) was added into the cell culture during PMA stimulation. Intracellular staining of INFγ-FITC (BD) was performed using Cytofix/Cytoperm™ (BD) in accordance with the manufacturer’s instructions. FACS Canto II (Becton-Dickinson, USA) and Flowjo (Tree Star, Inc.) were used to collect and analyze the data. *Inflammatory cytokine/chemokine assay:* the plasma samples collected after density-gradient centrifugation with Ficoll-Paque PLUS (GE Healthcare) were investigated for the presence of 13 human inflammatory cytokines/chemokines using the LEGENDplex^TM^ bead-based immunoassays (BioLegend) according to the manufacturer’s instruction. Using a total of 13 bead populations (Bead ID) distinguished by size and internal fluorescent dye, the panel allowed simultaneous detection of IL1b, IL12p70, IL18, MCP-1, IL33, INFa2, TNFα, IL10, IL6, IL8, IL17A, and IL23 in a single sample. Standard curves for each analytic and the samples were raced in duplicate. Data analysis was done using LEGENDplex^TM^ Data Analysis Software.

### 2.5. Statistical Analysis

Collected data were processed using Prism 5 (GraphPad Software, San Diego, CA, USA). Statistical significance was evaluated using a one-way ANOVA with Dunnett’s post-test, considering significant *p* < 0.05 (*), *p* < 0.01 (**) and *p* < 0.001 (***). The correlation profile “R^2^” between two different parameters was calculated by the Spearman rank correlation test.

## 3. Results

### 3.1. Evidence for X-Chromosome Inactivation Age-Independent in X-CGD Carriers

Fifteen females related to X-CGD patients referred to our Center for patients’ follow-up were enrolled and investigated for the presence of mutations in the *CYBB* gene and for the capacity of their granulocytes to correctly perform the *respiratory burst* (Table 1). Twelve females among all (S1–S12), including two related females S7 and S12 (mother and sister of an X-CGD patient, respectively), carried mutations in the *CYBB* gene, including missense mutations (50%), large deletions (41%), and duplications (9%) [13,16] The remaining three females (S13–S15) without mutations were considered healthy. In order to better interpret the data, X-CGD carriers were divided into a young adults (age: 20–39 years, n = 4) group (A), and a middle-aged adults (age: 40–59 years, *n* = 8) group (B).

**Figure 1:**
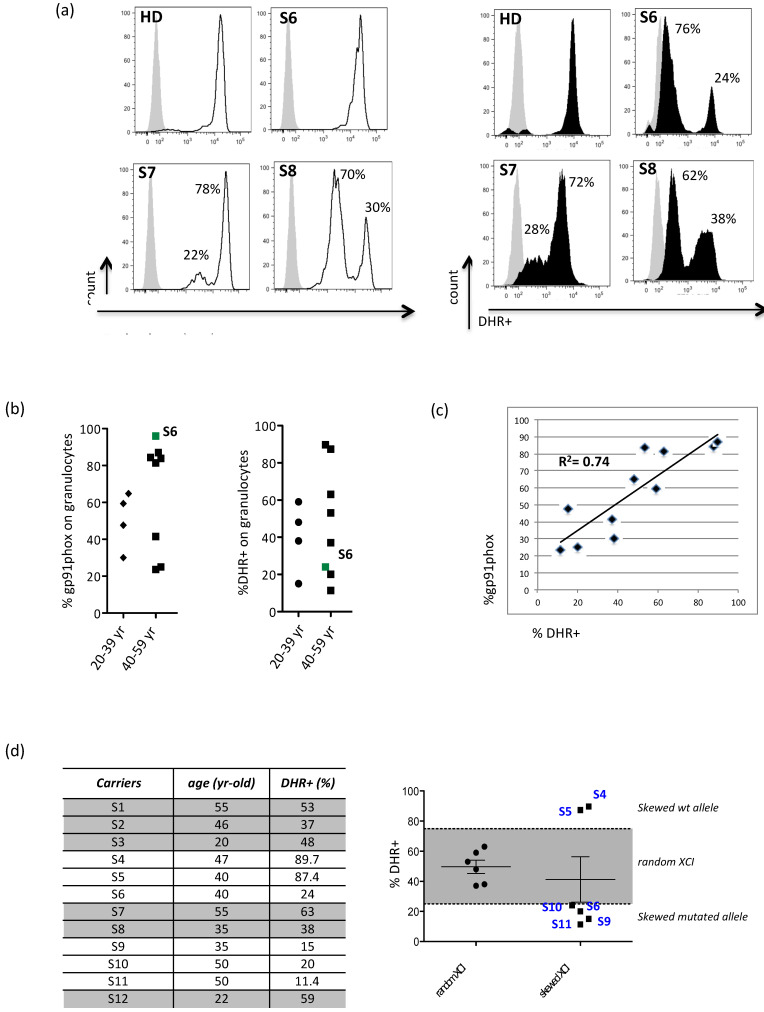
Evidence for X-chromosome inactivation age-independent in X-CGD carriers. (**a**) Representative FACS plots showing the gp91phox protein expression and NADPH oxidase function (DHR+) upon PMA stimulation of granulocytes from S6 (X91/X91+), S7 (X91/X91°), S8 (X91/X91°) carriers; (**b**) graphs showing the % of gp91phox protein and DHR+ cells evaluated in all carriers stratified by age. Each point shows a single X-CGD subject. The indicated S6 carrier is the only one with an X91/X91+ protein profile; (**c**) Correlation profile (*R*^2^ = 0.74) estimated between the % of gp91phox protein and the % of DHR+ value in carriers with X91/X91 (Spearman rank correlation test); (**d**) left panel shows the % of DHR+ evaluated by age. On the right, each point shows a single female subject; the grey zone indicates the random-XCI (from 25% to 75%), and the white external area indicates skewed XCI (wt gene: S4, S5; mutated gene: S6, S9, S10, S11).

All carriers, except S6, presented two different cell subsets of granulocytes, negative or positive for the gp91phox expression (Figure 1a,b). Reduced level of gp91phox protein did not prevent the NADPH oxidase from generating ROSs after PMA stimulation (Figure 1a,b) and opsonized E. coli (data not shown) although at lower levels than normal. The rate of oxidation was calculated as the percentage of granulocytes expressing DHR+ and the obtained results ranging from 15% to 90%. We found no significant correlation between the age and the percentage of wt protein (*R*^2^ = 0.002) or the percentage of DHR+ cells (*R*^2^ = 0.003), but as expected, we found a strong correlation (*R*^2^ = 0.74192) between the percentage of gp91phox-wt protein and granulocyte producing ROSs (Figure 1c). The S6 carrier represents the exception because although her granulocytes seem to exclusively express the mutated form of the gp91phox protein, they are able to produce a small amount of ROS and a low percentage of DHR+ cells. Probably, in this carrier, the mutated and the wt protein share the same mean-florescent intensity (MFI), being indistinguishable from one another. Finally, considering the percentage of DHR+ cells, ranged from 25 to 75 as the percentage benchmark in accordance with a random XCI, we found that independent from age (young adult: *R*^2^ = 0.01 and middle-aged adult: *R*^2^ = 0.001), 50% of carriers had a random XCI and the remaining 50% had a skewed XCI. Interestingly, about 66% of the carriers with skewed XCI (*n* = 4; B group: S6, S10, S11, and A group: S9) mainly expressed the mutated form of the *CYBB* gene (Figure 1d).

### 3.2. Clinical Manifestations and Inflammatory Cytokine/Chemokine Profiling in X-CGD Carriers

**Figure 2:**
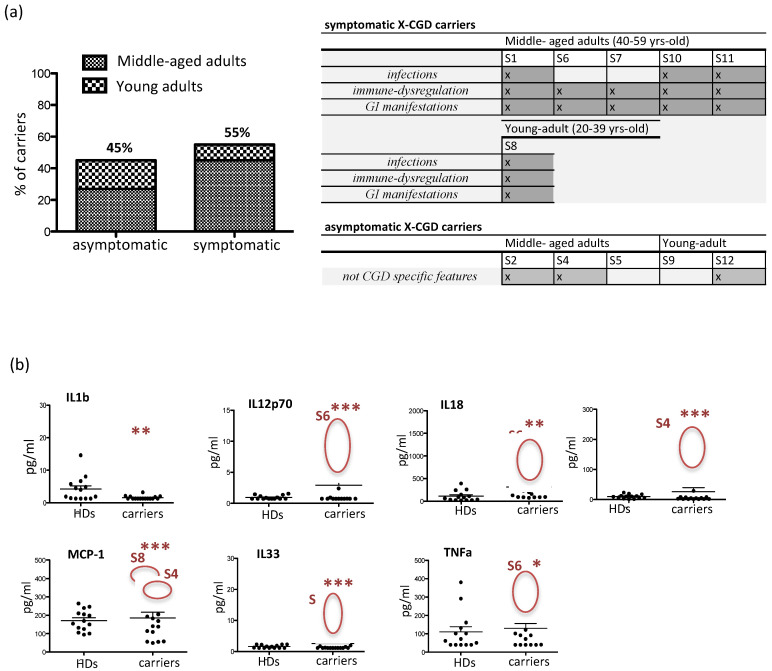
Clinical manifestations in X-CGD. (**a**) Left panel shows the percentage of symptomatic and asymptomatic carriers; right panel illustrates distribution of different clinical features according to age; (**b**) plasmatic pro-inflammatory profile. Each point shows a single female subject. Circles include X-CGD carriers that deviate from average, thus resulting statistically significant. Statistical test used for the data analysis was one-way ANOVA with Dunnett’s post-test considering * *p* < 0.05, ** *p* < 0.01 and *** *p* < 0.001.

A clinical diagnostic interview was conducted with eleven X-CGD carriers to collect information on symptoms and clinical findings in order to have a clear chart review of all the manifestations in the enrolled subjects. In particular, we investigated the presence of four groups of symptoms, including *infections*, *immune dysregulation*, *gastro-intestinal (GI) manifestations,* and not CGD-specific symptoms. The 55% of carriers (*n* = 6), including five middle-aged adult carriers (S1, S6, S7, S10, and S11) and one young adult carrier (S8), were symptomatic and manifested all investigated symptoms, but mainly (67%) immune dysregulation and GI manifestations (Figure 2a). The remaining 45% (*n* = 5) of X-CGD carriers represented the asymptomatic subjects that were constituted of three carriers belonging to the middle-aged adult group (S2, S4, and S5) and two carriers belonging to the young adult carrier group (S9 and S12). An inflammatory profile of the plasma samples obtained from carriers was investigated and compared to healthy donors (*n* = 10) for the presence of 13 human inflammatory cytokines/chemokines, including IL1b, IL12p70, IL18, MCP-1, IL33, INFa2, TNFα, IL10, IL6, IL8, IL17A, and IL23 (Figure 2b). In general, all plasmatic cytokines were normally represented, with the exception of IL1b that was statistically reduced (*p* < 0.05) (carriers 4.1 ± 1 pg/mL vs. HD 1.5 ± 0.1 pg/mL). However some X-CGD carriers had significant augmented level of IL12p70 (S6 9 ± 1.4 pg/mL vs. HD 0.95 ± 0.08 pg/mL; *p* < 0.001), IL18 (S6 794 ± 115 pg/mL vs. HD 111 ± 30 pg/mL; *p* < 0.01), INFa (S4 167 ± 11 pg/mL vs. HD 9.6 ± 1.8 pg/mL; *p* < 0.001), MCP-1 (S4 and S8 371 ± 24 pg/mL vs. HD 171 ± 15 pg/mL; *p* < 0.001), IL33 (S8 11.7 ± 4.8 pg/mL vs. HD 1.6 ± 0.13 pg/mL; *p* < 0.001) and TNFα (S6 263 ± 29 pg/mL vs. HD 110 ± 28 pg/mL; *p* < 0.05).

### 3.3. CD56bright NK Cell Are Expanded in Older X-CGD Carriers

A phenotypic study performed on peripheral blood mononuclear cells (PBMC) revealed a general normal distribution of all immune subsets in our cohort of X-CGD carriers (Table 2). CD4+ and CD8+ T cell and *classical* (CD16-CD14+), *non-classical* (CD16+CD14−) and *intermediate* (CD16+CD14bright and CD16+CD14low) subsets of monocytes resulted typically expressed. We found a small increment in CD19+ B cell percentage that resulted statistically significant (*p* < 0.01) in middle-aged adult carriers (young aged adult 12.6 ± 0.4 and middle-aged adult 15.7 ± 2.7 vs. HD 8.3 ± 1). Additionally, natural killer T-like (NKT)- and NK-cells were normally represented, although a statistically significant (*p* < 0.01) expansion toward NKbright (CD56+CD16-) was observed in middle-aged adult carriers (middle-aged adult 34 ± 6.2 vs. HD 17.6 ± 1.9). Then, the frequencies of CD56bright cells were used to stratify the results in consideration of the symptomatology, and we did not find any differences between both groups of carriers and the HDs (data not shown). Moreover, we investigated the correlation between the frequencies of CD56bright or CD56dim cells and the frequencies of DHR+ cells, and we did not find any correlation (*R*^2^ = 5 × 10^−5^ or *R*^2^ = 0.04, respectively) (data not shown).

### 3.4. The Residual Production of ROS (% DHR+ Cells) Correlates in X-CGD Carriers with the Reduced Percentage of Both CD8 Cells and INFg-CD4 Expressing Cells

**Figure 3:**
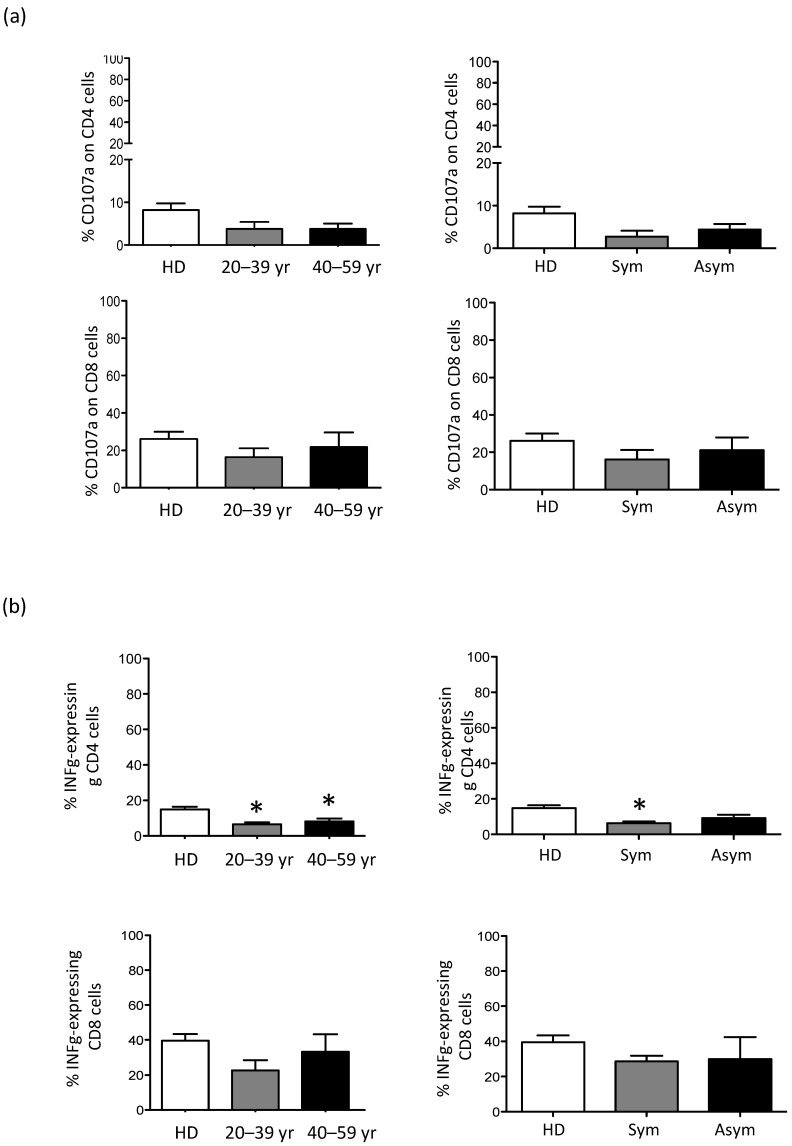
Functional analysis of CD4+ and CD8+ T cells. (**a**) Evaluation of CD4+ and CD8+ T cells capacity to upregulate the CD107a on cellular surface or (**b**) to produce INFγ after stimulation. Results from X-CGD carriers are showed in consideration of age and symptomatology and compared with *n* = 28HDs. Statistical significance was evaluated using one-way ANOVA with Dunnett’s post-test, considering: * *p* < 0.05.

**Figure 4:**
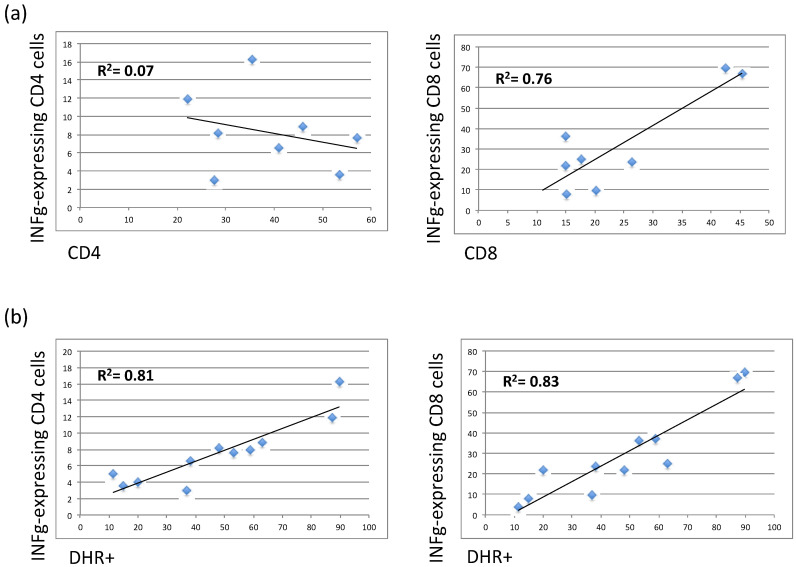
ROS production correlated with the reduced percentage in both CD8 cells and INFg-CD4 expressing cells. Correlation profiles (*Spearman rank correlation test)* were calculated: (**a**) % of CD4 cells vs. INFγ-expressing CD4 cells and % of CD8 cells vs. INFγ-expressing CD8 cells; (**b**) % of DHR+ vs. INFγ-expressing CD4 or CD8 cells.

To assess the functionality of NK, NK T-like, and T cells, stimulated PBMC were investigated for their ability to produce INFγ and to upregulate CD107a, a cell surface marker of immune cell activation and cytotoxic degranulation. The results showed that both NK and NK T-like populations in X-CGD carriers did not have any functional defects in maintaining their cytotoxicity (data not shown). CD4+ T cells showed the tendency to have reduced CD107a upregulation, whereas CD8 T cells showed the tendency to produce a reduced level of INFγ (Figure 3a,b). The percentage of INFγ by CD4 cells was significantly reduced (*p* < 0.05) in both young adult and middle-aged X-CGD carriers (young adult 6.5 ± 1.3 and middle-aged adult 8.1 ± 1.7 vs. HD 14.9 ± 1.5). Considering both symptomatic and asymptomatic carriers’ groups, we found a reinforced difference (*p* < 0.05) between HDs and the symptomatic group (Figure 3b). Moreover, differently from the CD8 cells, we found a lack of correlation (*R*^2^ = 0.07) between the percentage of total CD4+ cells and the levels of INFγ-CD4 expressing cells, suggesting that a functional defect in this subset is independent on CD4 T cell frequencies (Figure 4a). Additionally, the percentages of DHR+ cells were strongly correlated with the percentage of INFγ produced by CD8 (*R*^2^ = 0.83) or CD4 cells (*R*^2^ = 0.81) (Figure 4b).

## 4. Discussion

X-linked CGD is the most frequent form of CGD with a severe clinical phenotype characterized by bacterial and fungal infections and inflammatory complications, including granuloma, IBD, and autoimmunity. According to the Lyon hypothesis and the mechanism of random dosage compensation of X-linked genes in females, X-CGD carriers should be healthy because about half of their cells carrying the X chromosome express the wild-type gene [17]. Usually, the amount of ROS produced by X-CGD carriers is enough to protect them from significant infections, but in the last years, several studies described carriers presenting with a ‘CGD-like’ phenotype, suggesting that a skewed XCI determined the prevalence of the mutated non-functional allele [18]. Up to now, it is clear that even if granulocytes, primarily neutrophils, represent the central defective immune component of CGD, other non-phagocytic leukocytes operate in the pathogenesis of the disease [19]. Moreover, the imbalance between the production of ROSs and their elimination by protective cellular mechanisms can lead to cellular damage and to aging processes that could influence the pathogenesis of the disease [20,21,22]. In this study, we report the molecular, clinical, and immunological characterization of twelve X-CGD carriers previously genetically characterized because of their relation to our X-CGD cohort of patients in order to investigate new features underlying the pathogenesis of the disease. We found that all carriers except one (S6) had two populations of granulocytes, one positive and the other one negative for the gp91phox expression/function. Our data showed a variable gp91phox protein expression and NADPH oxidase function independent of the carriers’ age. As expected, a strong correlation (*R*^2^ = 0.74192) between the percentage of gp91phox+ cells and DHR+ cells was found with the exception of the S6 carrier, the only one in which the mutated protein is expressed, and it is indistinguishable from the correct protein form. In this carrier, only 24% of granulocytes were able to produce ROS demonstrating the prevalence of a mutated allele with respect to that wt and suggesting the importance of considering this finding for proper clinical management. Moreover, we found that 50% of carriers presented a skewed inactivation independent of age, and about 66% of these carried the mutated form of the *CYBB* gene, confirming the prevalence of a mutated allele over wt.

In the last year, several studies investigated the link between the skewed XCI related to age, the degree of ROS produced by functioning neutrophils, and the manifestations of disease in X-CGD carriers [7,10,15,23]. In our study, we described the 55% of carriers with a CGD-like phenotype, but the majority of them (67%) presented immune dysregulation and GI manifestations. This group was very heterogeneous with respect to age (S8_young adult and S1, S6, S7, S10, S11_middle-aged adult) and XCI (S6, S10, S11_skewed vs. mutated gene and S1, S7, S8_random), highlighting the difficulty to establish the criteria for classifying and well defining the clinical aspects of X-CGD carriers. The same heterogeneity was found in the asymptomatic group, including young (S9 and S12) and middle-aged (S2, S4, S5) adult carriers. Interestingly this last group comprised the only two carriers, S4 and S5, belonging to the middle-aged adult group that had a prevalence of wt gene and the S9 that had a prevalence of mutated gene. These results underlined the variability of carriers’ phenotypes that is probably influenced by other pathogenic mechanisms. Moreover, the increased levels of pro-inflammatory plasmatic cytokines, including TNFα and IL33 [24,25,26], MCP-1 [27], IL12p70 and IL18 [28], and IFNa2 [29] support the involvement of other factors such as age, genetic predisposition and environmental causes that could be contributing to the clinical manifestations.

The phenotypic analysis of PB immune cells in our relatively small but well-characterized carrier cohort revealed an increment in CD19+ B cells and CD56bright NK cells (CD56+CD16−) that resulted statistically significant in older carriers (*p* < 0.01). The CD56bright population is considered to be the immature precursors of CD56dim cells, and they usually represent 10–20% of total NK cells [29]. Although an expansion of peripheral CD56bright cells has been reported in several diseases characterized by autoimmunity and/or inflammation [30,31], we did not identify any differences in the frequencies of CD56bright cells in symptomatic and asymptomatic carriers compared to HD. Moreover, there was not any correlation between this subset and the percentage of DHR+ cells. Additionally, CD4+ T cells had an impaired CD107a uptake capacity, but both CD4+ and CD8+ T cell subsets produced a reduced level of INFγ. Notably, the frequency of INFγ-expressing CD4 cells was significantly reduced (*p* < 0.05) in both young adult and middle-aged X-CGD carriers, and this data was confirmed by the analysis of the symptomatic carriers (*p* < 0.05). Although the low level of INFγ in CD8 cells was correlated (*R*^2^ = 0,76) to their frequencies into the peripheral blood, this is not the case with CD4 cells (*R*^2^ = 0,14), suggesting an inherent defect in this cellular subset. In support of these findings, we demonstrated, in a previously published work, that an impaired T cell compartment in X-CGD patients, that was more evident in older patients in which the inability of the immune system to solve the infections results in chronic immune dysregulation, probably leads to aging of the immune system [32,33]. Finally, the interesting strong correlation between the frequencies of DHR+ cells and those of INFγ-expressing CD8 cells (*R*^2^ = 0.86) and INFγ-expressing CD4 cells (*R*^2^ = 0.85) highlighted a role for ROSs in the functioning and cytokines production of T cells [34].

## 5. Conclusions

In conclusion, our results reveal novel abnormalities in immune cells that might have a role in promoting the inflammatory status of X-CGD carriers despite the small number of investigated subjects and the collection of samples at a single time point. Moreover, the study of these aspects in a larger cohort of carriers could provide new insights to be considered during clinical management.

## Figures and Tables

**Table 1:** Molecular Diagnosis of X-CGD carrier or HD status. Molecular and protein analysis revealed 12 X-CGD carriers (S1–S12) and 3 healthy (S13–S15).

Female Analysed (Range of Age)	Mutation in *CYBB* Gene	Protein Expression (wt/mut)	X-Linked Carrier Status
S1 (B)	581bp del	X91/ X91°	carrier
S2 (B)	c.925G>A/p.E309K	X91/**X91°**	carrier
S3 (A)	c.469C>T /p.R157X.	X91/**X91°**	carrier
S4 (B)	c.252G>A/p.A84	X91/**X91°**	carrier
S5 (B)	c.742dupA p.Ile248AsnFsX36	X91/**X91°**	carrier
S6 (B)	c.1531T>G /p.Y511D	X91/**X91+**	carrier
S7 (B)	1637 Mbp del + McLeod	X91/**X91°**	carrier
S8 (A)	32,72 Kb del	X91/**X91°**	carrier
S9 (A)	c.736C>T/p.Q246X	X91/**X91°**	carrier
S10 (B)	c.1357T>A/p.W453R	X91/**X91°**	carrier
S11 (B)	ex9-11del	X91/**X91°**	carrier
S12 (A)	1637 Mbp del + McLeod	X91/**X91°**	carrier
S13	-	X91/X91	HD
S14	-	X91/X91	HD
S15	-	X91/X91	HD
HDs (*n* = 25)	-	X91/X91	HD

**Table 2:** Immune phenotype of X-CGD carriers (*n* = 12) compared to HDs (*n* = 28).

	HD	Carriers 20–39 yr	Carriers 40–59 yr
*gated on lymphocytes*	(%)	(%)	(%)
T cells (CD3+)	72.6 ± 1.9	72 ± 3.4	63 ± 8
CD4 Tcells (CD3+CD4+)	45.7 ± 3.1	44 ± 5.6	41 ±5.3
CD8 T cells (CD3+CD8+)	26.5 ± 1.4	19 ± 2.6	28 ± 6.1
B cells (CD19+)	8.3 ± 1	12.6 ± 0.4	15.7 ± 2.7 **
NKT cells (CD3+CD56+)	4.8 ± 0.6	4.3 ± 0.4	8.3 ± 2.7
NK cells (CD3-CD56+)	9.3 ± 1.4	5.8 ± 0.3	6.8 ± 1.3
*gated on NK cells*			
NK bright (CD16-)	17.6 ± 1.9	20 ± 8.3	34 ± 6.2 **
NK dim (CD16+)	74.5 ± 3.14	68 ± 4.8	61 ± 4
CD16+CD14- (non-classical)	11.5 ± 2.1	nd	8.4 ±3.1
CD16+CD14low (late-intermediate)	0.6 ± 0.19	nd	1.2 ± 0.4
CD16+CD14bright (early-intermediate)	0.7 ± 0.22	nd	0.7 ± 0.1
CD16-CD14+ (classical)	17.6 ± 2.6	nd	17.71 ± 4.7

* *p* < 0.05 and **p < 0.01 statistically significant; nd = not determined.

## Data Availability

Data sharing is available.

## Abbreviations

Chronic granulomatous disease (CGD)
Nicotinamide dinucleotide phosphate (NADPH)
X-linked CGD (X-CGD)
Reactive oxygen species (ROSs)
Inflammatory bowel disease (IBD)
X-chromosome inactivation (XCI)
Dihydrorhodamine 123 (DHR)
Peripheral blood mononuclear cell (PBMC)
Phorbol 12-myristate 13-acetate (PMA)
Healthy Donor (HD)
Wild type (wt)
